# IGHV1 usage is associated with lymphadenopathy and aggressive disease in the TCL1 mouse model for chronic lymphocytic leukemia

**DOI:** 10.1038/s41598-025-23109-5

**Published:** 2025-11-10

**Authors:** Stephan Drothler, Christian Scherhäufl, Carina Suete, Thomas Parigger, Franz Josef Gassner, Lisa Pleyer, Alexander Egle, Richard Greil, Roland Geisberger, Nadja Zaborsky

**Affiliations:** 1 Department of Internal Medicine III with Haematology, Medical Oncology, Haemostaseology, Infectiology and Rheumatology, Oncologic Center, Paracelsus Medical University, Müllner Hauptstr. 48, 5020 Salzburg, Austria; 2 Salzburg Cancer Research Institute - Laboratory for Immunological and Molecular Cancer Research (LIMCR), Salzburg, Austria; 3 Department of Biosciences, Paris-Lodron-University Salzburg, Salzburg, Austria; 4 Cancer Cluster Salzburg, Salzburg, Austria

**Keywords:** CLL, TCL1 mouse model, Cancer microenvironment, Lymphadenopathy, Translational research, Chronic lymphocytic leukaemia, Cancer microenvironment, Cancer models, Tumour immunology

## Abstract

TCL1 mice are the most commonly used preclinical model for chronic lymphocytic leukemia (CLL), a B-cell malignancy characterized by clonal CD5^+^ B-lymphocyte accumulation. B-cell receptor (BCR) sequencing identifies two important risk markers, immunoglobulin heavy chain variable region (IGHV) mutational status and receptor stereotypy. Despite its clinical relevance in patients, comprehensive examinations of endogenous BCR repertoires in TCL1 mice remain limited. We analysed BCR repertoires of 85 TCL1 mice, primarily comprising CLL clones using IGHV1 and IGHV11 (27.3 and 49.1% of CLL clones, respectively). Interestingly, TCL1 mice with dominant IGHV1 CLL clones showed significantly higher levels of CD4^+^ T-cells, and increased exhaustion levels (PD-1) on splenic CD8^+^ T-cells compared to IGHV11 CLL clones. Cancer related pathways (p53, MTORC and KRAS) were distinctly regulated in IGHV1 CLL clones. These clones occurred more frequently in female mice, characterized by short survival times (hazard ratio 2.6). Additionally, mice with dominant IGHV1 CLL clones displayed an almost twofold inguinal lymph node enlargement. In conclusion, we identified molecular, phenotypical and immunological differences between IGHV1 and IGHV11 CLL clones, which are key to consider for preclinical studies using the TCL1 mouse model. Furthermore, our data suggests that IGHV1 CLL clones model the nodal form of human CLL.

**Supplementary Information:**

The online version contains supplementary material available at 10.1038/s41598-025-23109-5.

## Introduction

Chronic lymphocytic leukemia (CLL) is a B-cell malignancy with highly diverse clinical courses portrayed by a heterogeneous genetic landscape, with seminal features like del11q, del17p, del13q and tri12. Unmutated immunoglobulin heavy chain variable region (IGHV) genes of the B-cell receptor (BCR) are strongly associated with an aggressive disease course and different prognostic impacts of recurring gene mutations^1,2^. Moreover, one third of patients can be grouped based on their (stereotyped) BCRs, which are characterized by varying aggressiveness^3^. CLL can emerge as a leukemic or lymphoma version, termed small lymphocytic lymphoma (SLL)^4^.

Since its introduction in 2002, the T-Cell Leukemia/Lymphoma 1 A (TCL1) transgenic mouse has served as a highly researched model for the aggressive, unmutated form of CLL^5^. Similar to human disease, TCL1 mice show stereotyped complementarity determining region 3 (CDR3) of the BCR, whereby specific sequences have been linked to B1 cells, a self-renewing CD5^+^ B-cell subset, typically secreting polyreactive natural antibodies^6–8^.

Studies characterizing the BCR repertoire of TCL1 mice have typically utilized small sample sizes, leading to limited statistical power^5,8^. Since BCR sequencing is a standard procedure for CLL diagnosis and disease monitoring, yielding highly relevant clinical information, we sequenced a large cohort of primary TCL1 mice (*n* = 85), to gain a deeper understanding of VDJ gene usage and CDR3 characteristics of CLL clones. Subsequently, we analysed survival, T-cell skewing, transcriptional profiles and lymphadenopathy based on IGHV subgroups of dominant CLL clones.

## Results

### Cohort description

**Fig. 1:**
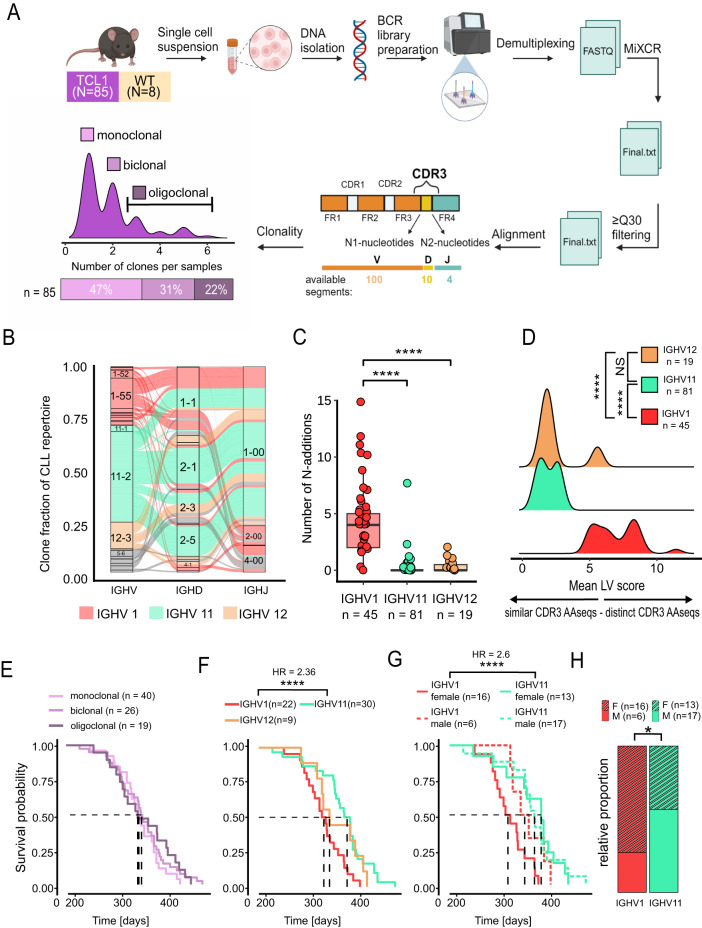
Study design, clonality, diversity and survival analyses. (**A**) Processing pipeline from organ sample to individual clones. BCR clones ≥ 5% were defined as CLL clones, whereas clones < 5% were considered as B-cells and consequently labelled as miscellaneous (“Misc”) (see Methods). (**B**) Alluvial plot of IGHV, IGHD, and IGHJ gene usage in TCL1 mouse derived CLL clones (*n* = 166), labelled respective IGH genes. (**C**) N-nucleotide insertion (summed N1 and N2 nucleotides) for different CLL clones IGHV1 (27%), IGHV11 (49%) and IGHV12 (11%). Wilcoxon test was used for significance computation. (**D**) Sequence similarity (LV score) of aaCDR3 regions of all IGHV1, IGHV11 and IGHV12 clones. LV values were adjusted for clone fraction and 100 randomly selected aaCDR3 sequences were compared (see Methods). (**E**) Kaplan-Meier survival curves based on clonality, including monoclonal (*n* = 40), biclonal (*n* = 26) and oligoclonal (*n* = 19) TCL1 mice. (**F**) Kaplan-Meier survival curves, grouped by IGHV-genes IGHV11 (*n* = 30), IGHV12 (*n* = 9) and IGHV1 (*n* = 22), with a dominant clone > 50%. Individual strata were compared pairwise using log-rank tests with Benjamini-Hochberg false discovery adjustment for multiple comparisons. Hazard ratio (HR) was calculated for significant groups. (**G**) Kaplan-Meier survival curves, of IGHV11 and IGHV1 stratified by sex, with a dominant clone > 50% (IGHV11 female *n* = 13, IGHV11 male *n* = 17, IGHV1 female *n* = 16, IGHV1 male *n* = 6). Individual strata were compared pairwise using log-rank tests with Benjamini-Hochberg false discovery adjustment for multiple comparisons. Hazard ratio (HR) was calculated for significant groups. (**H**) Bar chart summarizing sex distribution of IGHV1 and IGHV11 mice, Fisher’s Exact Test was used to compare differences in sex distribution.

BCR repertoires of 85 TCL1 mice were analysed (52% male; 48% female) (Fig. 1A & Supp. Table 1). Consistent with literature, male TCL1 mice survived significantly longer than female TCL1 mice (median_male_: 355 days; 95% CI: 344 to 388 vs. median_female_: 337 days; 95% CI: 313 to 365; *p* = 0.0088) (Supp. Fig. 1A)^9^.

Individual clones were differentiated by their CDR3 amino acid sequences. A representative wild type (WT) cohort (*n* = 8; median age = 308 days; 62% female) was assembled, to establish a biologically informed threshold to define a CLL clone. The ten most abundant clones in WT mice ranged from 1.3% to 3% clone fraction (median 1.5%) (Supp. Table 2). To set an adequate cut-off, we additionally considered an established threshold for human CLL, ranging from 2.5 to 5% clone fraction^10^. Therefore, we defined BCR clones ≥ 5% as CLL clones, whereas clones < 5% were considered as B-cells and consequently labelled as miscellaneous (“Misc”).

The mice were grouped based on their respective numbers of distinct CLL clones. The relative majority of TCL1 mice (47%) showed a monoclonal disease phenotype, followed by biclonal tumors with a share of 31%, while oligoclonal (3–10 clones) phenotypes accounted for 22% (Fig. 1A).

In order to capture dynamics of BCR repertoires during tumor growth, 14 TCL1 and 14 WT mice were sampled by peripheral blood draws (TCL1: *n* = 70; WT: *n* = 73) on a monthly basis (from 2 to 17 months), until humane endpoint (Supp. Tables 3 & 4). The normalized Herfindahl Hirschman Index (HHI), a diversity metric^11,12^, (monoclonal repertoire = 1) was used to assess changes in BCR repertoire variety over time.

Expectedly, the HHI of TCL1 mice was positively correlated with age (R^2^ = 0.39; *p* < 0.001), showing decreased BCR repertoire diversity compared to WT mice (R^2^ < 0.01; *p* = 0.90) (Supp. Fig. 1B).

### CLL cells show restricted VDJ usage and distinct N-nucleotide insertions in TCL1 mice

We observed three prevalently used IGHV subgroups, IGHV1 (*n* = 45, 27.3%), IGHV11 (*n* = 81, 49.1%) and IGHV12 (*n* = 19, 11.5%), accounting for 87.9% of all CLL clones (*n* = 166) (Fig. 1B). All identified CLL clones had an unmutated IGHV mutation status. Junction analysis of the CDR3 region was performed, using the international immunogenetics information system^13^. Strong differences in N-nucleotide insertions between IGHV1 (median: 4; interquartile range (IQR): 2–5), IGHV11 (median: 0; IQR: 0–0) and IGHV12 clones were found (median: 0; IQR: 0-0.5) (IGHV1 vs. IGHV11 11: *p* < 0.001 and IGHV1 vs. IGHV12: *p* < 0.001; IGHV11 vs. IGHV12: *p* = 0.02) (Fig. 1C).

Furthermore, we compared CDR3 sequence diversity of IGHV1, IGHV11 and IGHV12 CLL clones. We observed decreased Levenshtein distances (sequence diversity) in IGHV11 (median: 1.75; IQR: 1.3–2.6) and IGHV12 (median: 1.89; IQR: 1.8–2.1) CDR3 sequences compared to IGHV1 (median: 6.74; IQR: 5.7–8.2) (IGHV1 vs. IGHV11 *p* < 0.001; IGHV1 vs. IGHV12 *p* < 0.001; IGHV11 vs. IGHV12 *p* = 0.53) (Fig. 1D), caused by occurrence of invariant BCRs sequences amongst IGHV11 and IGHV12 clones (Supp. Fig. 1C). Furthermore, the invariant BCRs of IGHV11 and IGHV12 clones are associated with conserved antigen recognition of phosphatidylcholine^8,14^.

### Dominant IGHV1 clones are associated with an aggressive disease course in female TCL1 mice

CLL clonality was not associated with overall survival (Fig. 1E).

For the remaining survival analyses, mice with a dominant clone of at least 50% clone fraction were used, to minimize potential clonal crossover effects from bi- and oligoclonal TCL1 mice. IGHV1 mice had a significantly shorter overall survival than IGHV11 mice (median_IGHV1_: 322 days; 95% CI: 303 to 352 vs. median _IGHV11_: 371 days; 95% CI: 348 to 383; Benjamini-Hochberg (BH) adjusted log rank: *p* = 0.002), but not IGHV12 (median_IGHV12_: 334 days; lower 95% CI: 318; BH-adjusted log rank: *p* = 0.1) (Fig. 1F). IGHV11 and IGHV12 mice did not differ significantly in terms of survival (BH-adjusted log rank: *p* = 0.37).

Additionally, we analysed IGHV1 and IGHV11 mice based on sex. Interestingly, female mice with a dominant IGHV1 CLL clone had the shortest overall survival (median_IGHV1 female_: 308 days; 95% CI: 286 to 364) of all groups. Male IGHV11 and IGHV1 mice showed no survival difference (median_IGHV1 male_: 344 days; lower 95% CI: 318 vs. median _IGHV11 male_: 364 days; 95% CI: 346 to 389; *p* = 0.51). Overall survival was significantly lower for female mice with a dominant IGHV1 CLL clone versus a dominant IGHV11 (median_IGHV11 female_: 378 days; lower 95% CI: 342; *p* = 0.002) (Fig. 1G). IGHV11 clones were more frequently found in male mice (57% males), whereas IGHV1 clones had higher occurrence in female mice (73% females) (Fishers exact estimate = 3.4; *p* = 0.049) (Fig. 1H).

### IGHV1 and IGHV11 CLL clones display distinct pathway deregulation and lineage profiles in TCL1 mice

**Fig. 2:**
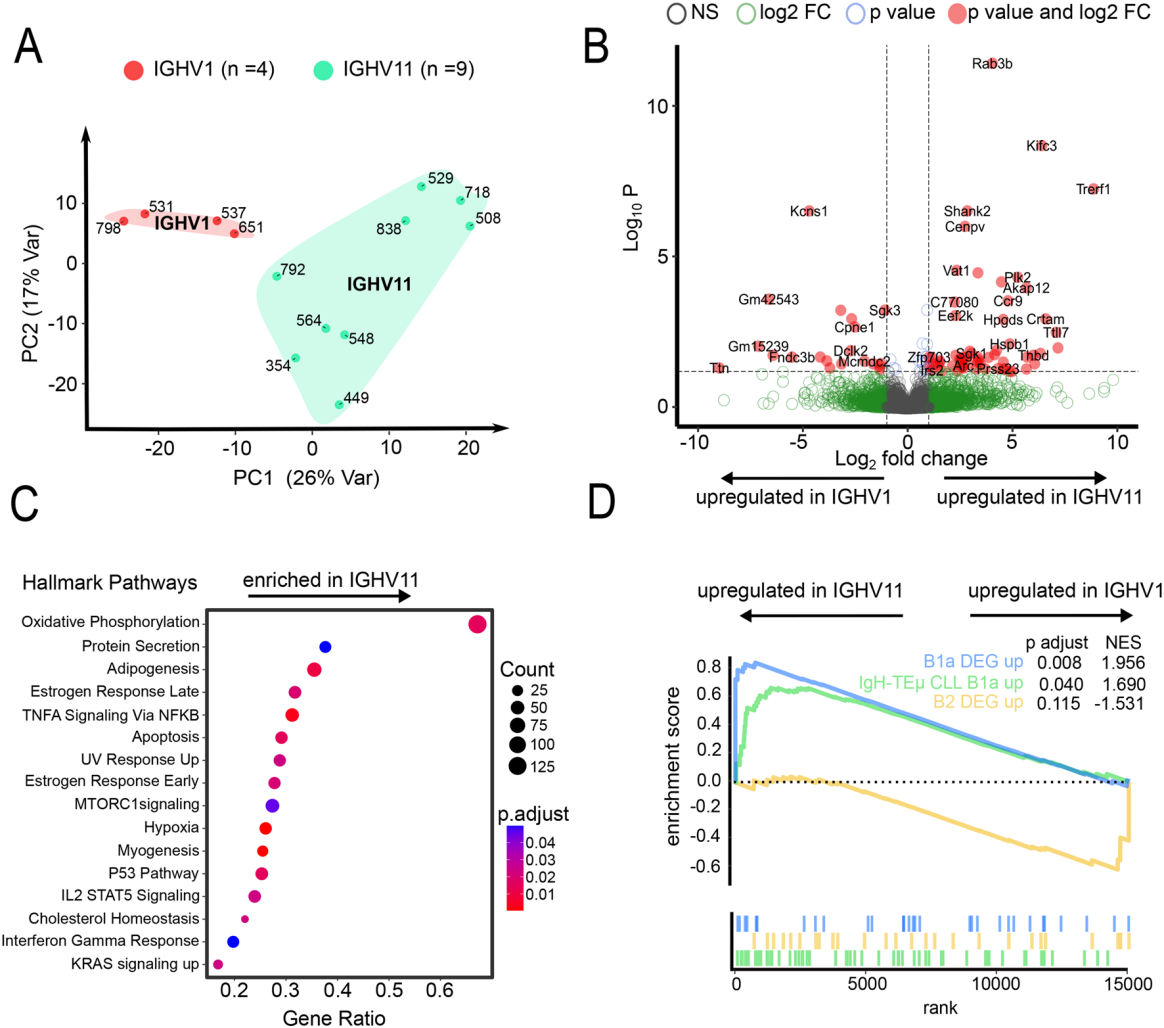
Transcriptome analysis of TCL1 mice. (**A**) Principle component analysis of transcriptome analysis from IGHV11 (*n* = 9) and IGHV1 (*n* = 4) mice from sorted CD5^+^CD19^+^ splenocytes. (**B**) Volcano plot indicating differentially expressed genes of IGHV11 (*n* = 9) and IGHV1 (*n* = 4) mice from sorted CD5^+^CD19^+^ splenocytes. Genes up- or downregulated with a fold change ≥ 2 and an FDR < 0.05 are depicted in red. (**C**) Enriched MSigDB Hallmark pathways between IGHV11 (*n* = 9) and IGHV1 (*n* = 4) mice from sorted CD5^+^CD19^+^ splenocytes. Only significantly enriched pathways are depicted (padj. < 0.05). (**D**) Gene set enrichment analysis of three gene sets, each tested IGHV11 versus IGHV1. B1a and B2 specific gene sets were defined by Mabbot et al.^15^. IgH-TEµ CLL B1a gene set was published by Singh et al.^16^.

To further study biological disparities underlying survival differences between IGHV1 and IGHV11 mice, we analysed transcriptomes, using bulk RNA-Seq, of sorted splenic CD5^+^CD19^+^ cells from TCL1 mice (IGHV1: *n* = 4, all female; IGHV11: *n* = 9, male *n* = 5, female *n* = 4, clone fraction of dominant clone: IGHV1 ≥ 79%; IGHV11 ≥ 95%). Principal component analysis, excluding all IGH, IGK and IGL genes, revealed distinct transcriptional a profiles (Fig. 2A). In total, 106 significantly deregulated genes (fold change > 1) were identified (Fig. 2B). Gene set enrichment analysis (GSEA) indicated different pathway regulation between the two groups in important cancer associated pathways (p53, MTOR and KRAS), possibly contributing to the observed survival difference. Analysis of individual components of the oxidative phosphorylation machinery revealed an orchestrated deregulation of multiple components, leading to significant enrichment of the pathway with a leading edge of 63% of all genes (114 out of 181 genes; NES 1.67; q-value 0.019) (Fig. 2C).

IGHV11 clones display typical CDR3 sequences associated with B1a cells, whereas IGHV1 clones lack those^7^. GSEA was performed to determine transcriptional imprints of different B-cell subtypes still preserved after malignant transformation. We used two published gene sets for GSEA, characterizing B1a- and B2- B-cells^15^. IGHV11 clones showed a significant B1a gene signature (*p* = 0.008; normalized enrichment score (NES) = 1.96) (Fig. 2D). While IGHV1 clones did not significantly match any of the gene sets, they resembled conventional B2 cells the most (*p* = 0.115, NES = 1.531), however a more precise phenotypic characterization would require epigenetic analysis. In addition, IGHV11 clones from our TCL1 mice showed a transcription pattern similar to a published gene set originating from IGHV11 of an alternative CLL mouse model (IgH-TEµ mice) (*p* = 0.04, NES = 1.69)^16^ (Fig. 2D). Sex-stratified transcriptional profiling in mice with dominant IGHV11 CLL clones (*n* = 4 females; *n* = 5 males) identified seven differentially expressed genes, but was not performed in mice with dominant IGHV1 CLL clones, as only female TCL1 mice were available in this group. (Supp. Table 7)^17^.

### Different T-cell repertoire skewing based on CLL subsets in TCL1 mice

It has previously been shown in the IgH-TEµ model that IGHV11 tumor development is less T-cell dependent than non-IGHV11 tumors^16^. Therefore, we aimed to investigate whether IGHV11 versus IGHV1 CLL subsets differentially affect T-cell skewing in TCL1 mice. 14 cryopreserved TCL1 SPL samples, with a dominant CLL clone > = 77% (IGHV1: *n* = 8; IGHV11: *n* = 6; clone fraction: IGHV1 = 77–99%; IGHV11 = 95–99%), were used for flow cytometry analysis (Supp. Table 5).

**Fig. 3:**
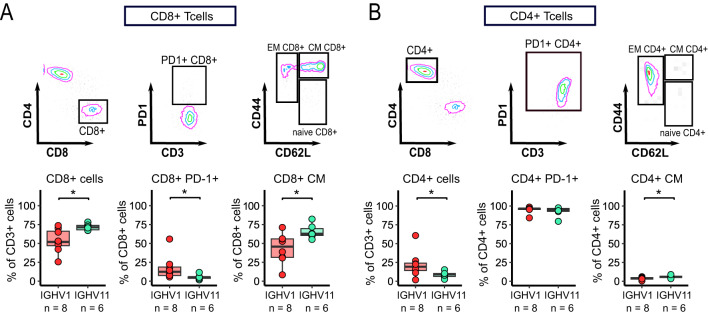
Flow cytometric analysis of TCL1 mice (**A**,**B**) Representative gating strategy for CD8^+^/CD4^+^ T-cell subsets (PD-1^+^, CM, EM and naive) and flow cytometry results of SPL from mice with dominant IGHV11 (*n* = 6) and IGHV1 (*n* = 8) CLL clones. Wilcoxon test was used to compare IGHV11 and IGHV1 data.

Mice with a dominant IGHV11 clone showed significantly higher CD8^+^ T-cell fractions among CD3^+^ T-cells compared to mice with a dominant IGHV1 clone (median: IGHV11: 71.4%; IQR: 68.1–73.9%; IGHV1: 52.0%; IQR: 47-66.3%; *p* = 0.02), (Fig. 3A). In contrast, mice with a dominant IGHV1 clone showed significantly higher fractions of PD-1 expressing CD8^+^ T-cells (median: IGHV11: 5.28%; IQR: 3.8–5.7%; IGHV1: 12.5%; IQR: 7.5–18.7%; *p* = 0.013) (Fig. 3A). PD-1 median fluorescence intensity levels of CD4^+^ and CD8^+^ T-cells subsets did not differ significantly between mice with dominant IGHV1 and IGHV11 CLL clones (Supp. Fig. 1D, Supp. Table 5). In addition, we further analysed CD8^+^ T-cell compartments with regard to their naïve/memory and activation phenotype. Interestingly, we observed significantly higher CD8^+^ central memory (CM) fractions (IGHV11: 62.8%; IQR: 60.6–69.6%; IGHV1: 45.8%; IQR: 31.6–56%; *p* = 0.02) in the IGHV11 cohort compared to IGHV1 (Fig. 3A). Increased fractions of activated T-cells and effector memory (EM) CD8^+^ T-cells were found in IGHV1 mice, however not at statistically significant levels (Supp. Fig. 1E).

Skewing towards a central memory phenotype was also detected in the CD4^+^ T-cell compartment of IGHV11 mice, albeit to a lesser extent (median: IGHV11: 5.67%; IQR: 4.9–6.7%; IGHV1: 4.1%; IQR: 2.7–4.7%;

*p* = 0.043). The high fraction of PD-1^+^CD4^+^ T-cells was comparable in both cohorts (median: IGHV11: 95%; IQR: 92.9–96.8%; IGHV1: 96.6%; IQR: 95.4–96.8%, *p* = 0.49) (Fig. 3B). Similarly, fractions of EM, naïve and activated T-cells were not significantly different between IGHV1 and IGHV11 (Supp. Fig. 1F). When stratifying the data by sex only, we found higher levels of naïve CD8^+^ and CD4^+^ T-cells in male mice compared to female mice (median: female: 0.6%; IQR: 0.3–1.3%; males: 3.2%; IQR: 2–4%; *p* = 0.017; Supp. Fig. 2A,B). When comparing T-cell skewing based on IGHV and sex we observed a significantly higher frequency of EM CD4^+^ T-cells in female vs. male mice with dominant IGHV1 CLL clones (median: IGHV1 female: 94.8%; IQR: 93.3–95.6%; IGHV1 males: 88.8%; IQR: 77.6–89.4%; *p* = 0.036), while levels of naïve CD4^+^ T-cells were significantly lower (median: IGHV1 female: 0.7%; IQR: 0.6–1.9%; IGHV1 males: 4.5%; IQR: 3.6–14%; *p* = 0.036; Supp. Fig. 2C). CD8^+^ T- cells subsets (naïve, EM, CM, PD-1^+^, CD69^+^) were comparable when grouped by IGHV and sex (Supp. Fig. 2D).

### IGHV1 CLL clones show more lymphadenopathy than IGHV11 CLL clones in TCL1 mice

**Fig. 4:**
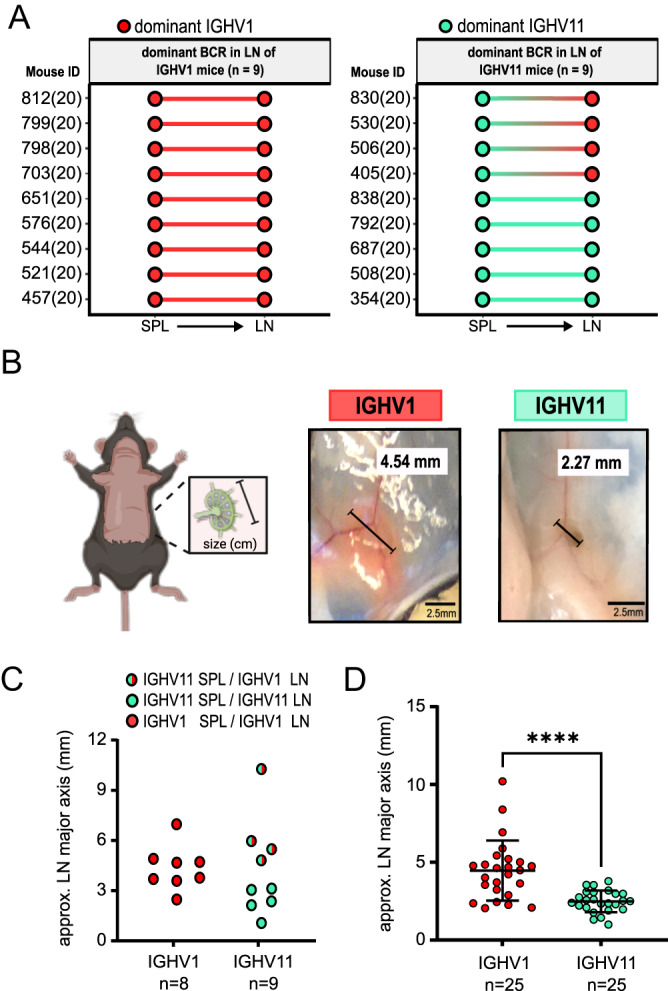
LN migration of IGHV11 and IGHV1 clones. (**A**) Paired dot plot comparing the dominant clone of matched SPL (*n* = 18; IGHV11 *n* = 9; IGHV1 *n* = 9) and LN (*n* = 18) samples. (**B**) Representative pictures of LNs from mice with a dominant IGHV11 (ID: 813) and IGHV1 (ID: 537) clone. (**C**) Dot plot comparing LN size, and dominant clone in SPL and LN of mice with analysed LN B-cell repertoire of IGHV11 (*n* = 9) and IGHV1 (*n* = 8, LN size of one mouse not measurable). (**D**) Dot plot displaying LN size based on BCR repertoire of LN if available or the initially sequenced organ of IGHV11 (*n* = 25) and IGHV1 (*n* = 25) mice. Wilcoxon test was used to compare IGHV11 and IGHV1 data.

Due to the observed differences between IGHV1 and IGHV11 mice, we characterized disparities in disease phenotypes by assessing lymphadenopathy, a key clinical parameter in human CLL. 18 cryopreserved LN samples were used for BCR sequencing (IGHV1: *n* = 9, IGHV11: *n* = 9). 14 out of 18 mice showed identical IGHV subgroups of dominant clones in SPL and LN. Four mice showed differences in IGHV subgroups of dominant clones across organs. Strikingly, these four mice showed a dominant IGHV11 CLL clone in SPL but a dominant IGHV1 CLL clone in LN (Fig. 4A). This led us to examine inguinal LN size, as a correlate for CLL infiltration, at endpoint (Fig. 4B). Remarkably, these four mice had the largest LNs of all nine analysed IGHV11 mice (Fig. 4C). Subsequent analysis of LN size from IGHV1 (*n* = 25) and IGHV11 (*n* = 25) mice revealed significantly larger LNs of IGHV1 mice (median LN size: IGHV1: 4.5 mm; IQR: 3.1–5.2 mm; IGHV11: 2.6 mm; IQR: 2.1–3.1 mm, *p* < 0.001) (Fig. 4D).

To investigate lymph node infiltration and validate the presence of CLL cells in lymphoid tissue, we performed further analysis of BCR sequencing data from lymph nodes (*n* = 18). Across these samples, 40 CLL clones were identified (≥ 5% BCR clone fraction), with a median clonal fraction of 74%. 16 CLL clones expressed IGHV1 and 16 CLL clones expressed IGHV11 (40% of all CLL clones, respectively). We compared the clone fractions of the dominant CLL clones, grouped by IGHV1 (*n* = 13) and IGHV11 (*n* = 5). Higher clonal fractions were observed for IGHV1 CLL clones compared to IGHV11 (median: IGHV1: 79%; IQR: 57–97%; IGHV11: 48%; IQR: 45–71%), although the difference did not reach statistical significance (*p* = 0.12; Supp. Fig. 2E). BCR repertoires in lymph nodes with dominant IGHV1 CLL clones were more frequently monoclonal, whereas repertoires with dominant IGHV11 CLL clones were exclusively biclonal (Fisher’s exact test, *p* < 0.04; Supp. Fig. 2F). When comparing occurrence of all CLL clones, IGHV1 clones showed a significantly higher median clone fraction compared to IGHV11 clones (median: IGHV1: 73%; IQR: 45–96%; IGHV11: 28%; IQR: 8–44%; *p* < 0.0019; Supp. Fig. 2G). These findings further indicate the infiltration of CLL clones into lymphoid tissues and suggest a preferential migration of IGHV1-expressing CLL clones into lymph nodes.

## Discussion

In this study, we linked molecular attributes of BCRs from CLL clones with clinical and pathobiological features in 85 TCL1 mice, a model for aggressive CLL. This study presents novel findings on survival differences, distinct transcriptomes, T-cell skewing, lymph node infiltration and organ preference of different CLL clones based on IGHV subgroups. We provide statistically robust analyses of IGHV subgroup distribution, nucleotide insertion rates, and CDR3 length, expanding current literature^8,18,19^. Similar to human CLL, we detected bi- and oligoclonal tumors, albeit at a higher frequency (TCL1 mice: 53% vs. patients: 2–5%)^20^, presumably caused by human TCL1 oncogene expression in all murine IgM^+^ cells^5^. CLL clonality (mono-, bi-, oligoclonal) was not associated with overall survival.

Dominant IGHV1 CLL clones, linked to enlarged LNs, are more prevalent in female mice and associated with reduced overall survival rates compared to females with dominant IGHV11 CLL clones. Jaksic et al., examined lymphatic and leukemic CLL variants of 341 patients. The authors found significantly shorter survival, only in female patients who suffered from lymphatic disease forms compared to more leukemic variants, which is in line with our mouse data^21^.

Gene expression profiling revealed distinct transcriptional profiles of murine IGHV1 and IGHV11 CLL clones. Closer analysis of genes upregulated in IGHV1 CLL clones identified several candidates potentially contributing to the decreased overall survival. Notably, increased expression of doublecortin-like kinase 2 (DCLK2), a serine/threonine-protein kinase, has previously been linked to reduced overall survival in CLL patients^22^. In addition, we identified genes associated with aggressive disease forms in CLL or other tumor entities, including serum- and glucocorticoid-inducible protein kinase 3 (SGK3), a regulator of the PI3K pathway^23–25^ and LIM domain only 7 (LMO7), which has been implicated in multiple cancer entities^26^.

Differentially expressed genes manifested in a set of deregulated hallmark pathways, including various cancer-associated pathways (p53, MTORC1, KRAS), indicating distinct pathway regulation patterns. We performed GSEA using published B1a gene sets, revealing an imprinted B1a transcriptional profile in IGHV11 CLL clones^15^. Further, IGHV11 clones showed high upregulation of genes relevant for the oxidative phosphorylation machinery, a pathway linked to B1a cells^27^. Enhanced oxidative phosphorylation correlates with adverse prognostic markers, including ZAP-70 expression and unmutated IGHV status, which is driven by BCR and PI3K signaling in CLL patients^29^. Furthermore, increased TNF-α receptor expression is associated with aggressive disease phenotypes in CLL^30^. Notably, these pathways are upregulated in IGHV11 CLL clones, which are associated with longer survival. The shorter survival of IGHV1 CLL clones may be explained by their preferential lymph node homing, where they likely receive activating stimuli from the environment, driving their proliferation and protecting CLL cells from apoptosis^28,29^.

We compared our findings with those reported by Zaborsky et al. Notably, in that study, all TCL1 mice, harboring a dominant IGHV11 CLL clone (ID 212, ID 221, ID 347, ID D22; *n* = 4) exhibited mutations (stop gain, non-frameshift deletions and non-synonymous) in genes associated with the AKT/mTOR pathway, including PTEN, PIK3R1, PIK3CA, ITGA7, KRAS and EGF^18^. Consistent with our pathway enrichment analysis, these findings suggest altered mTOR activity in IGHV11 mice. As the mTOR signaling pathway is influenced by both mutational alterations and transcriptional deregulation in mice harboring a dominant IGHV11 CLL clone, these findings might suggest that the mTOR pathway may play a more prominent role in IGHV11 CLL clones compared to IGHV1 CLL clones.

We identified three mice (ID 568, ID 701, ID 746) in our TCL1 cohort exhibiting a dominant IGHV12 clone, which harbors a CDR3 region with known low affinity phosphatidylcholine-binding capacity, as shown by Iacovelli et al.^30^. Given that this receptor is typically linked to B1 cells, these findings raise the possibility that IGHV12 CLL clones may originate from a B1 cell lineage^31^.

Singh et al. have previously shown in a different CLL mouse model (IgH-TEµ) that mice lacking germinal centers predominantly develop IGHV11 tumors while mice with activated T-cells have increased occurrence of IGHV1 tumors^16^. We showed distinct CD4^+^/CD8^+^ T-cell ratios, T-cell subset skewing and CLL migration to LNs based on IGHV subgroups in TCL1 mice. These findings point to a higher dependency of IGHV1 CLL clones on T-cells and distinct microenvironmental requirements. Furthermore, the observed differential skewing of T-cell subsets, particularly the significant difference in PD-1 expression on CD8^+^ T-cells based on the IGHV usage, may have implications for the efficacy of immune checkpoint inhibitors. These findings should be considered in the design of future preclinical studies evaluating immunotherapeutic approaches.

In SLL patients, negative prognostic factors such as unmutated IGHV, CD38, ZAP70, CD49d, trisomy 12 and *NOTCH1* mutations are observed more frequently compared to CLL, leading to short TFTT^28,32–40^. However, an association of nodal phenotypes with particular IGHV usage is less well documented, probably due to the low number of SLL cases analysed. While Daudignon et al. reported a lower frequency of IGHV1 usage in SLL compared to CLL (IGHV1: SLL 12%, CLL 20%), others reported an overrepresentation of IGHV1 in SLL (Groenen et al.)^41,42^. To the best of our knowledge, we are the first to identify divergent dominant clones within the SPL and LNs of TCL1 mice. This observation raises important questions regarding organ-specific clonal expansion, which warrants further investigation in CLL patients. Several studies have already documented the presence of multiple CLL clones in the peripheral blood of CLL patients, which might have potential relevance in a clinical context^43,44^.

Lymph nodes provide a protective niche for CLL cells, where they are exposed to pro-survival signals that enhance their resistance to both spontaneous and drug-induced apoptosis, mediated through CCL19 and CCL21^28^. Consequently, CLL clones with different IGHV subgroups in TCL1 transgenic mice may probably exhibit differential responses to therapeutic interventions, which may have translational implications.

Interestingly, CLL clones utilizing IGHV1 or IGHV11 are associated with distinct survival outcomes, transcriptional profiles, and dependence on different microenvironmental signals. Differential prognosis has been reported according to IGHV mutational status in patients^1^; however, though patients with mutated IGHV generally have better prediction on chemo-immunotherapy, both subgroups show improved response rates to targeted therapies such as BTK inhibition^45^. Therefore, it is likely that in the TCL1 mouse model BTK inhibitor efficacy is independent of IGHV usage, however, a differential response to future immune therapeutic strategies based on IGHV usage cannot be excluded.

Due to vast molecular and phenotypical differences between IGHV1 and IGHV11 CLL clones and the popularity of this murine model in preclinical studies for CLL we urge to implement analysis of IGHV subgroups as a standard specification in studies using TCL1 mice. Our study provides evidence for previously unknown intertumoral differences in lymphoadenopathic disease presentation in TCL1 mice, modelling a hallmark feature of SLL and advanced-stage CLL.

## Methods

### Mice

TCL1 transgenic mice were obtained from Carlo Croce (Kimmel Cancer Center, Philadelphia, USA) and backcrossed on a C57BL/6J background. Genotyping of TCL1 transgenic mice (C57BL/6J) was performed as previously described^46^. Mouse experiments were approved by the Austrian animal ethics committee and performed according to their guidelines (BMWF 66.012/0009-II/3b/2012, 20901-TGV/52/11-2012, BMBWF-66.012/0002-V/3b/2018 and BMBWF 2023 − 0.644.528) and adhere to the ARRIVE guidelines. Inclusion criteria and humane endpoints were defined by a tumor load in the peripheral blood ≥ 70%, increased abdominal size and/or signs of weakness. Mice were constantly monitored for signs of illness and sacrificed at humane endpoint by CO2 suffocation, when moribund, in line with the guidelines of Austrian animal ethics committee and the guidelines of the American Veterinary Medical Association (AMVA). After sacrificing mice, SPL and LNs were removed and homogenized, and splenocytes were treated with ACK for 10 min at room temperature to lyse erythrocytes. Peritoneal cavity was flushed with PBS. Both femurs were flushed with 5 ml PBS to isolate bone marrow. Cells were frozen in heat inactivated FBS (Gibco, Billings, MT, USA) with 10% dimethyl sulfoxide (DMSO).

### B-cell receptor sequencing

67x SPL-, 14x heart blood-, 3x peritoneal cavity-, 18x lymph node - samples and 1x bone marrow were analysed. DNA was isolated using the DNeasy Blood and Tissue Kit (Qiagen, Hilden, Germany), including an RNAse digest. BCR sequencing libraries were prepared as described previously^46^. Tapestation Bioanalyzer (Agilent, Santa Clara, CA, USA) was used to determine quality of sequencing libraries, which were sequenced on a MiSeq platform (Illumina, San Diego, CA, USA). FASTQ files were analysed using MIXCR software (version 2.1.8, MiLaboratory, Sunnyvale, CA, USA)^47^, to define clones based on the amino acid sequence forming the CDR3 of the immunoglobulin heavy chain, starting from the conserved cysteine 104 in the IGHV and the fixed tryptophan/phenylalanine 118 in the VJ region. Clones with a quality score of < Q30 and/or non-functional mutations were excluded from the analysis. D-segments alignments and IGHV-identity were manually checked using IMGT^13^.

### Flow cytometry

Tumor load in mice was measured on a Cytoflex (Beckman Coulter, Brea, CA, USA) using following antibodies:

anti-mouse CD62L FITC (clone: MEL-14) (Invitrogen, Carlsbad, CA, USA), anti-mouse CD8 APC-H7 (clone: 53 − 6.7) (BD, Franklin Lakes, NJ, USA), anti-mouse CD69 PE (clone: H1.2F3), anti-mouse CD44 PC5.5 (clone: IM7), anti-mouse CD45 PC7 (clone: I3/2.3), anti-mouse CD3 APC (clone: 17 A2 ), anti-mouse CD5 Brilliant Violet 421 (clone: 53 − 7.3), anti-mouse CD19 Brilliant Violet 605 (clone: 6D5), anti-mouse CD4 Brilliant Violet 650 (clone: RM4-5), anti-mouse PD-1 Brilliant Violet 785 (clone: 29 F.1A12) (Biolegend, San Diego, CA, USA).

For flow cytometry of splenocytes, erythrocytes were lysed using ACK (5 min at RT), and subsequently washed with PBS. Flow cytometry staining was performed in PBS (15 min at RT). Bloodless organs were handled the same way, with exception of erythrocyte lysis. Cryopreserved samples were rested 5 h in 10 ml RPMI medium (Gibco, cat. no. 31870-025) supplemented with 10% FCS (Gibco, Billings, MT, USA), 1% Pen/Strep (Biowest, Nuaillé, FR ) and 1% L-Glut (Biowest, Nuaillé, FR) at 37 °C, before they were stained.

### Cell sorting

Cryopreserved cells were thawed in 10 ml of prewarmed (37 °C) RPMI medium (Gibco, cat. no. 31870-025) supplemented with 10% FCS (Gibco, Billings, MT, USA), 1% Pen/Strep (Biowest, Nuaillé, FR ) and 1% L-Glut (Biowest, Nuaillé, FR). Cells were centrifuged at 350 g for 5 min and washed once with 10 ml PBS (Gibco, Billings, MT, USA cat. no. 10010056) and filtered (30 μm, Sysmex, Goerlitz, Germany). Cell concentration and viability was determined using EVE automatic cell analyser (NanoEntek, Guro-gu, South Korea) or LUNA-FX7 automated cell counter (logos biosystems, Gyeonggi-do, South Korea). 14 samples were used for fluorescence-activated cell sorting using an ARIA III instrument (BD, Franklin Lakes, NJ, USA). 2.0 × 10^7^ – 2.2 × 10^7^ viable cells were stained for 20 min at room temperature in PBS, using the following antibodies: anti-mouse CD5 BV421 (clone: 53 − 7.3); anti-mouse CD4 FITC (clone: RM4-4); anti-mouse CD19 APC (clone: 6D5) (Biolegend, San Diego, CA, USA); anti-mouse CD8a APC-H7 (clone: 53 − 6.7) (BD, Franklin Lakes, NJ, USA). After staining, cells were washed once with 2 ml PBS, resuspended in sort buffer (PBS supplemented with 2% FCS and 0.025 M HEPES (Gibco, Billings, MT, USA, cat: 15630-080), and CD5^+^/CD19^+^ cells were sorted. Purity of sorted cell population was verified for all sorted samples, showing a consistently high purity of 96.2–99.9%.

### Transcriptome analysis

TCL1 CLL tumor cells were FACS sorted from mouse splenocytes according to the above-described cell sorting procedure and RNA was subsequently isolated using the RNeasy Mini Kit (Qiagen, Hilden, Germany). The quality of the RNA was assessed using the Tapestation Bioanalyzer showing RIN scores from 2.4 to 8.6. We used 50.6–200 ng of input RNA for rRNA depletion via the NEBNext rRNA Depletion Kit v2 (Human/Mouse/Rat) (NEB Ipswich, MA, USA; cat: E7405L) and sequencing libraries were prepared via the NEBNext Ultra II Directional RNA Library Kit for Illumina (NEB; cat: E7760S). Libraries were quality checked by Tapestation Bioanalyzer (Agilent, Santa Clara, USA) and subsequently sequenced (75 bp single-end) on a NextSeq 550 platform (Illumina, San Diego, CA, USA) with a 1% PhiX spike-in. To assess potential effects of low RIN on library quality, we correlated sample RIN values with three key RNA-seq quality metrics: total read counts (R^2^ = 0.0045), duplication rate (R^2^ = 0.0184), and the number of genes with non-zero counts (R^2^ = 0.0011). This analysis revealed no evidence of correlation between RIN and these quality metrics.

Raw sequencing reads were converted to fastq files and demultiplexed using the bcl2fastq conversion software v2.19.1.403 (Illumina, San Diego, CA, USA). The quality of the output fastq files was evaluated by the FastQC v0.11.9 software^48^. Fastq reads were then trimmed using trimmomatic v0.39^49^ before they were again quality checked by FastQC and aligned to the mouse reference genome (mm39) using the STAR Aligner v2.7.9a^50^. Aligned reads were then sorted and indexed using the samtools software v1.10^51^ and gene counts were calculated using the featureCounts software (subread-1.6.3 package)^52^. PCA analysis was performed using the R packages DESeq2 v3.17^53^ and PCAtools v2.16^54^ after excluding all Igh*, Igk* and Igl* genes since these are associated with sample classification. Male and female mice with dominant IGHV11 CLL clones were compared after excluding Y-chromosomal genes, since these are associated with sample classification. Differential gene expression (DE) analysis was then conducted using the R package DESeq2 v3.17: SizeFactors and Dispersions were calculated before applying the sex-adjusted Likelihood ratio test (design = ~ gender + group) by using the DESeq function. Volcano plots were drawn using the R package EnhancedVolcano v1.18.0^55^. Based on DE analysis, GSEA was performed using the fgsea package v4.3^56^ and various MSigDB gene signatures^57^ were retrieved through the msigdbr v7.5.1 package^58^ and additional gene sets were obtained from literature^15,16^. Enriched gene sets were displayed by the usage of the ClusterProfiler v 4.8.2 R package^59^. Transcriptome-based BCR repertoire analysis was conducted by the MiXCR software v4.3.0^47^ by applying the ‘*mixcr analyse rnaseq-cdr3*’ command.

### Lymph node size determination

Photos of mice with visible inguinal LNs, taken during organ harvest, were used to determine widest elongation of the cardial side inguinal LN. Size was measured using ImageJ 1.53c^60^, and an in-picture size reference, by a blinded operator. Random samples were cross-validated by a second blinded operator.

### Statistical analysis

Data processing, descriptive statistics, HHI, LV, survival analysis and frequentist comparisons between measures of centrality were performed and visualized with R studio (R Version 4.3.1) loaded with the following main packages: tidyverse (V2.0.0)^61^, rstatix (V 0.7.2)^62^, ggpmisc (V 0.5.5)^63^, diverse (V 0.1.5)^64^, survminer (V 0.4.9)^65^, survival (V 3.4.0)^66^, ggstatsplot (V 0.12.1)^67^, dlookr (V 0.6.2)^68^, ComplexHeatmap (2.16.0)^69^, stringdist (V 0.9.12)^70^, and ggalluvial (V 0.12.5)^71^ .

Clonal occurrences were normalized for LV distance calculation and alluvial plotting by adjusting the frequency of individual CDR3 sequences with the cumulated clone fraction trough multiplication.

Shapiro-Wilk test was used to determine normal distribution. Since no comparisons consisted of exclusively normally distributed groups, non-parametric Wilcoxon tests were used throughout the manuscript.

Medians are reported with the interquartile range (IQR: 25th percentile – 75th percentile). If sample size was sufficient, 95% confidence intervals (CI: lower – upper) were reported for survival analysis. Otherwise, only lower or upper bounds of the 95% CIs were reported.

## Supplementary Information

Below is the link to the electronic supplementary material.

Supplementary Material 1

Supplementary Material 2

## Data Availability

The datasets generated during and/or analysed during the current study are available in the SRA repository, BioProject ID: PRJNA1117729. http://www.ncbi.nlm.nih.gov/bioproject/1117729.
