# Supplementary material for: IGHV1 usage is associated with lymphadenopathy and aggressive disease in the TCL1 mouse model for chronic lymphocytic leukemia
Source: Sci Rep. 2025 Nov 10;15:39290. doi: 10.1038/s41598-025-23109-5 (PMC12603018; doi:10.1038/s41598-025-23109-5)
Supplement: Supplementary file 1 — Supplementary Material 1 [file 41598_2025_23109_MOESM1_ESM.pdf]

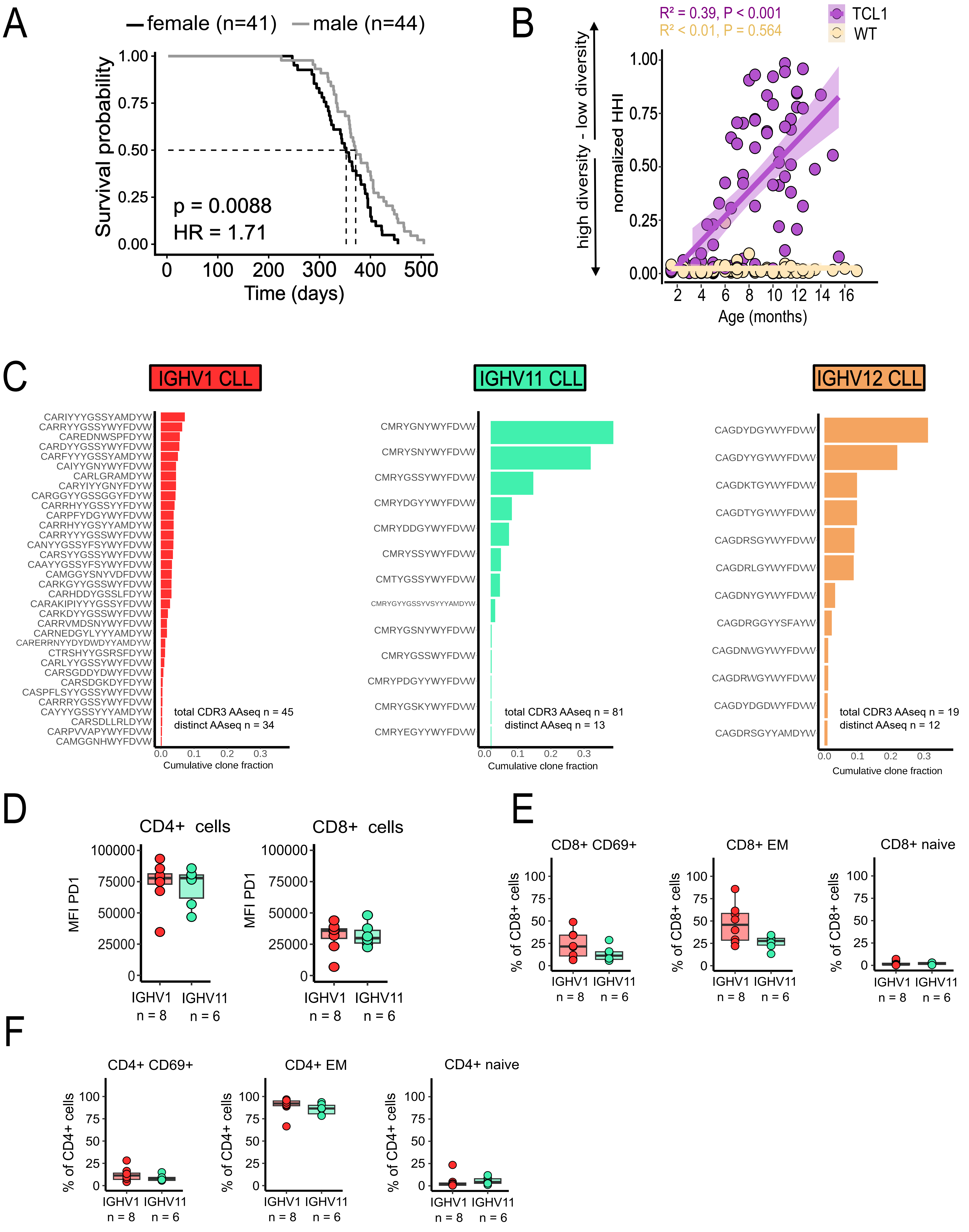

Supplement Figure 1. (A) Kaplan-Meier survival curves, grouped by sex (female mice: n =41; male mice: n = 44). **(B)** Scatter plot comparing changes of BCR-repertoire diversity in WT (n=73 samples) and TCL1 (n= 69 samples) mice over time. **(C)** Bar graph showing cohort specific cumulative clone fraction of distinct aaCDR3 sequences of IGHV groups (IGHV1 n = 45, IGHV11 n = 81, IGHV12 = 19). **(D)** Flow cytometric results of the median fluorescent intensity of PD -1 for $CD8^+$ and $CD4^+$ T-cells grouped by IGHV gene usage of the dominant CLL clone (IGHV1: n = 8; IGHV11: n = 6). Wilcoxon test was used to compare female and male data. **(E + F)** Flow cytometric results for $CD8^+$ and $CD4^+$ T-cell subsets ($CD69^+$, EM and naive) grouped by IGHV gene usage of the dominant CLL clone (IGHV11: n = 6; IGHV1: n = 8). Wilcoxon test was used to compare IGHV11 and IGHV1 data.

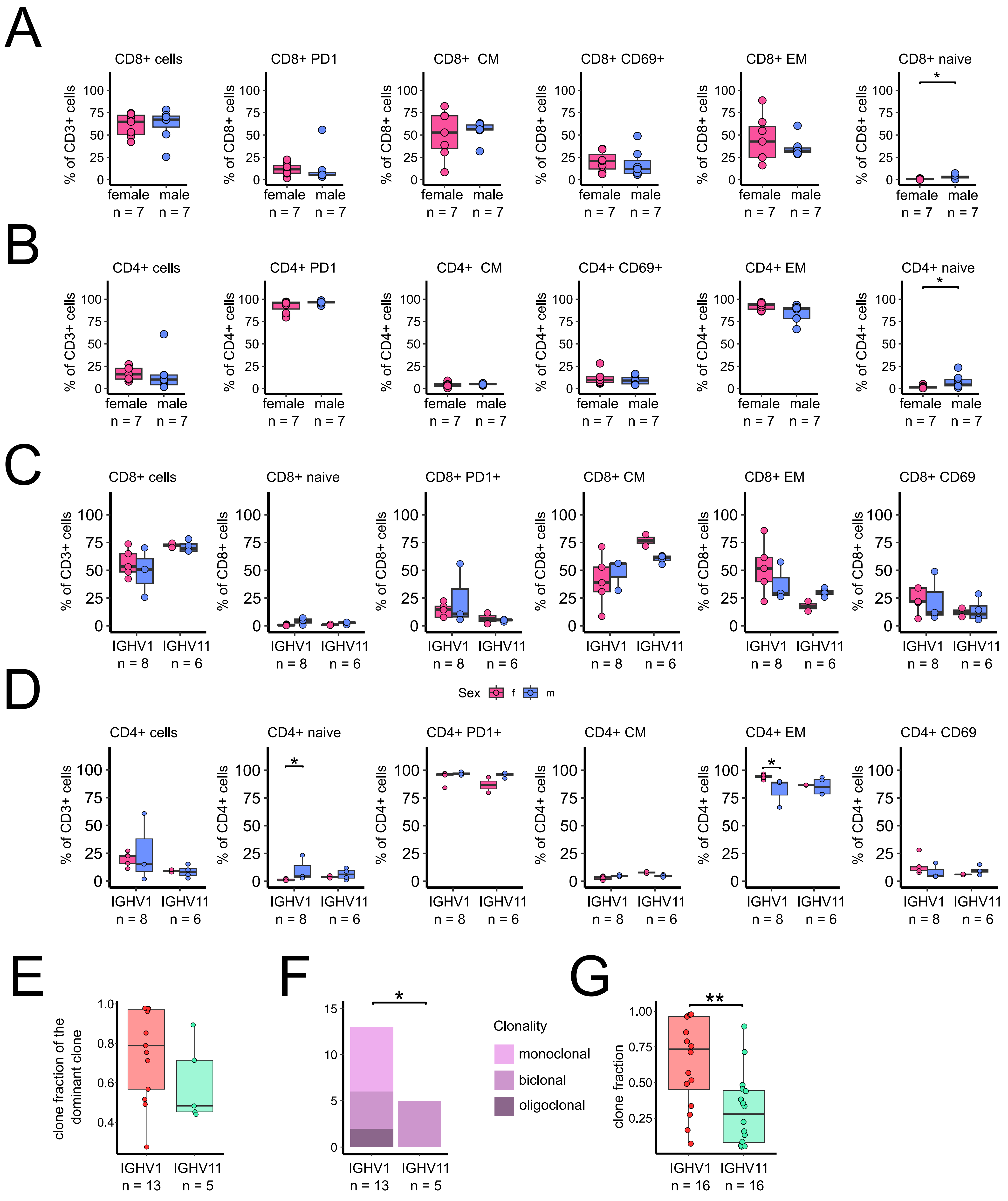

Supplement Figure 2. (A + B) Flow cytometric results for $CD8^+$ and $CD4^+$ T-cells and subsets ($CD69^+$, PD1, CM, EM and naive) grouped by sex (female: n = 7; male: n = 8). Wilcoxon test was used to compare female and male data. **(C + D)** Flow cytometric results for $CD4^+$ and $CD8^+$ T-cells and subsets ($CD69^+$, PD1, CM, EM and naive) grouped by IGHV gene usage of the dominant CLL clone and sex (IGHV1 female: n = 5; IGHV1 male: n = 3; IGHV11 female: n = 2; IGHV11 male: n = 4). Wilcoxon test was used to compare female and male data. **(E)** Comparison of clone fractions of dominant CLL clones in lymph nodes, grouped by IGHV1 (n = 13) and IGHV11 (n = 5). Wilcoxon test was used to compare IGHV1 and IGHV11 ($p = 0.12$). **(F)** Clonality of BCR repertoires in lymph node samples stratified by dominant IGHV usage (Fisher's exact test, $p < 0.04$). **(G)** Distribution of clonal fractions for all detected IGHV1 and IGHV11 CLL clones across lymph node samples. Wilcoxon test was used to compare clone fractions of IGHV1 and IGHV11 CLL clones ($p < 0.0019$).
